# The Diseases of Children

**Published:** 1890-03

**Authors:** 


*The Diseases of Children.* By HENRY ASHBY, M.D., M.R.C.P., and G. A. WRIGHT, B.A., M.B., F.R.C.S. Pp. 681. London: LONGMANS, GREEN & CO. 1889.

We have carefully and leisurely perused this book, comparing it with others on the same subject, and have come to the conclusion that it is the best work of moderate size at present in existence which can be placed in the hands of the English reader. The authors are evidently scientific men of a high order: their opportunities for clinical experience have been exceptionally great; they have, to the full, utilised their opportunities, and they have presented their experience and their knowledge in a simple, clear and straightforward manner, which commands at once credence and respect.

